# Comprehensive analysis of the *Spartina alterniflora WD40* gene family reveals the regulatory role of *SaTTG1* in plant development

**DOI:** 10.3389/fpls.2024.1390461

**Published:** 2024-05-28

**Authors:** Maogeng Yang, Shoukun Chen, Jiahui Geng, Shuqiang Gao, Shihua Chen, Huihui Li

**Affiliations:** ^1^ Key Laboratory of Plant Molecular & Developmental Biology, College of Life Sciences, Yantai University, Yantai, Shandong, China; ^2^ State Key Laboratory of Crop Gene Resources and Breeding, Institute of Crop Sciences, Chinese Academy of Agricultural Sciences (CAAS), Beijing, China; ^3^ Nanfan Research Institute, Chinese Academy of Agricultural Sciences (CAAS), Sanya, Hainan, China

**Keywords:** *Spartina alterniflora*, WD40, *TTG1*, flowering time, seed size

## Abstract

**Introduction:**

The *WD40* gene family, prevalent in eukaryotes, assumes diverse roles in cellular processes. *Spartina alterniflora*, a halophyte with exceptional salt tolerance, flood tolerance, reproduction, and diffusion ability, offers great potential for industrial applications and crop breeding analysis. The exploration of growth and development-related genes in this species offers immense potential for enhancing crop yield and environmental adaptability, particularly in industrialized plantations. However, the understanding of their role in regulating plant growth and development remains limited.

**Methods:**

In this study, we conducted a comprehensive analysis of *WD40* genes in *S. alterniflora* at the whole-genome level, delving into their characteristics such as physicochemical properties, phylogenetic relationships, gene architecture, and expression patterns. Additionally, we cloned the *TTG1* gene, a gene in plant growth and development across diverse species.

**Results:**

We identified a total of 582 WD40 proteins in the *S. alterniflora* genome, exhibiting an uneven distribution across chromosomes. Through phylogenetic analysis, we categorized the 582 SaWD40 proteins into 12 distinct clades. Examining the duplication patterns of *SaWD40* genes, we observed a predominant role of segmental duplication in their expansion. A substantial proportion of *SaWD40* gene duplication pairs underwent purifying selection through evolution. To explore the functional aspects, we selected *SaTTG1*, a homolog of *Arabidopsis TTG1*, for overexpression in Arabidopsis. Subcellular localization analysis revealed that the SaTTG1 protein localized in the nucleus and plasma membrane, exhibiting transcriptional activation in yeast cells. The overexpression of *SaTTG1* in *Arabidopsis* resulted in early flowering and increased seed size.

**Discussion:**

These outcomes significantly contribute to our understanding of WD40 gene functions in halophyte species. The findings not only serve as a valuable foundation for further investigations into *WD40* genes in halophyte but also offer insights into the molecular mechanisms governing plant development, offering potential avenues in molecular breeding.

## 1 Introduction

The WD40 protein, alternatively known as the WD repeat protein, comprises a regulatory superfamily that is ubiquitously found in eukaryotes (Jain and Pandey, 2018). This protein features multiple WD repeat motifs, each consisting of 44–60 amino acid residues (Mishra et al., 2012). These WD repeat motifs are characterized by a glycine histidine dipeptide at the N-terminus and a tryptophan aspartate dipeptide at the C-terminus (Smith et al., 1999). Folding of WD repeat motifs creates a four-stranded antiparallel β-sheet, stabilizing the WD40 protein fold through a robust hydrogen bonding network between N- and C-termini (Xu and Min, 2011). WD40 domains typically contain four to eight WD repeats, forming β-propeller structures (Stirnimann et al., 2010). WD40 proteins have been systematically identified across various plant genomes, with 237 in *Arabidopsis* (Van Nocker and Ludwig, 2003), 743 in wheat (Hu et al., 2018), and 191 in *Cucumis sativus* (Chen et al., 2023).

Accumulating evidence underscores the diverse roles played by WD40 proteins in various physiological and biochemical processes in plants. *TRANSPARENT TESTA GLABRA 1* (*TTG1*), an initially identified WD40 protein in *Arabidopsis*, serves as a component of the MYB-bHLH-WD40 complex, regulating anthocyanin biosynthesis in plants. This complex has been observed in diverse species such as rice (Sun et al., 2022), blueberry fruits (Zhao et al., 2019), *Ficus carica* (Fan et al., 2022), *Punica granatum* (Ben-Simhon et al., 2011), and *Camellia sinensis* (Liu et al., 2018). Furthermore, the overexpression of TTG-like gene *CsWD40* in tobacco significantly increased anthocyanin content in the transgenic plant’s petals (Liu et al., 2018). Additionally, Ehd5 acts as a positive regulator of rice flowering, providing insights into the molecular mechanisms underlying heading date (Zhang et al., 2023). Moreover, *Fvcpc2* plays a crucial role in regulating mushroom development and yield in *Flammulina velutipes* (Wu et al., 2020). These findings underscore the significance of WD40 proteins in plant growth and development.

*WD40* genes emerge as pivotal regulators in abiotic stress responses and hormonal signaling cascades. One notable instance is *GbLWD1*-like, a *WD40* gene from *Ginkgo biloba*, which enhances salt tolerance in transgenic *Populus* (Xin et al., 2021). *XIW1* (XPO1-interacting WD40 protein 1) positively influences the abscisic acid response in *Arabidopsis* (Xu et al., 2019). A mutation in *XIW1* results in the reduced induction of *ABI5* and ABA-responsive genes under salt treatment (Cai et al., 2020). Heterologous overexpression of *TaWD40D* in *Arabidopsis* significantly enhances tolerance to hormonal responses during seed germination and abiotic stresses during seedling development (Kong et al., 2015). *OsRACK1A*, regulated by the circadian rhythm, plays a role in regulating salt stress responses (Zhang et al., 2018). These findings underscore the pivotal role of *WD40* genes in plant stress tolerance and hormonal responses.

*Spartina alterniflora*, a halophyte thriving in coastal salt marshes, holds considerable economic value in coastal natural wetlands. Despite being an invasive species, this species produces more aboveground and underground biomass than local populations, making it a promising source of biochar (Liu et al., 2022), and a raw material for anaerobic digestion processes to produce biogas (Yang et al., 2009). Additionally, it has multiple applications such as livestock feed, fertilizer, and production of bio mineral liquids with various health benefits (Qin et al., 1998; Qiu et al., 2020; Zhou et al., 2023). The ideal ecological characteristics of biomass energy crops include C_4_ photosynthesis, long canopy duration, permanence, absence of pests or diseases, rapid spring growth, rigidity, and efficient water use (Raghu et al., 2006). *S. alterniflora* possesses most of these characteristics, making it an excellent candidate for industrial and energy development (Buhle et al., 2012). Therefore, exploring the excellent energy development genes of *S. alterniflora* is crucial for addressing energy security challenges.

*S. alterniflora* has potential for as biofuel feedstock, enhancing crop yield and environmental adaptability through the exploration of growth and development-related genes. However, limited research has focused on gene functional analysis in this species. Presently, reported studies on *S. alterniflora* genes have primarily focused on salt stress tolerance, with scarcely any research exploring the functions of its genome in plant growth and development. This gap in knowledge presents a significant opportunity for further exploration. In this investigation, we conducted a comprehensive analysis of *WD40* genes at the whole-genome level in *S. alterniflora*, exploring their characteristics, including physical and chemical properties, phylogenetic tree, gene structure, and expression patterns. Furthermore, we cloned the *TTG1* gene, which plays a pivotal role in plant growth and development across various species. Our aim is to decipher the functions of the *WD40* gene family by studying the functional significance of *TTG1*, as it may provide insights into the regulation of flowering and seed size, crucial processes that directly impact crop yield and quality. This study lies in its comprehensive analysis of *WD40* genes in *S. alterniflora*, particularly the cloning and functional exploration of the *TTG1* gene, which bridges the knowledge gap on the role of this species’ genome in plant growth and development.

## 2 Materials and methods

### 2.1 Identification of *WD40* genes in *S. alterniflora*

The genomic data, encompassing DNA, complete coding sequence (CDS), protein sequence, and positional annotations of *S. alterniflora*, were procured from our compiled genome. To identify WD40 proteins in *S. alterniflora*, we initially searched for sequences exhibiting the characteristic WD40 motif (PF00400) within the *S. alterniflora* protein sequences using HMMER 3.0. The putative SaWD40 proteins were validated using SMART (Letunic et al., 2021) and NCBI’s conserved domain database (Marchler-Bauer et al., 2015). Finally, only the proteins containing the WD40 repeat were retained for subsequent analysis.

To examine their physical attributes, all SaWD40 sequences were assessed using EXPASY to record the number of amino acids and molecular.

### 2.2 Classification and phylogenetic analysis of SaWD40s

To construct a phylogenetic tree for the SaWD40s, we first performed multiple sequence alignment of the full-length SaWD40 protein sequences using the T-COFFEE program (Magis et al., 2014). Subsequently, we employed the Neighbor-Joining (NJ) method in MEGA 7 to build the phylogenetic tree, with a Bootstrap value of 1000 and other default parameters (Kumar et al., 2016). Finally, we utilized Evolview v3 (Subramanian et al., 2019) to visualize the generated phylogenetic tree.

### 2.3 Gene duplication analysis

To analyze gene duplication events in *SaWD40* genes, we employed MCScanX (Wang et al., 2012) with default settings to identify various gene duplicates, including whole-genome and tandem duplications. The chromosomal locations and duplicated WD40 gene pairs were visualized using TBtools software (Chen et al., 2020). Evolutionary dynamics of these gene pairs were assessed by calculating *Ka/Ks* values using KaKs_Calculator v 2.0 (Zhang, 2022). The divergence time was determined using the formula T = *Ks*/2λ, where *Ks* signifies the synonymous substitutions per site, and λ represents the rate of divergence for nuclear genes in plants. For monocot plants, the adopted λ value fell within the range of 5.1–7.1×10^–9^ synonymous substitutions per site per year.

### 2.4 Plant materials, RNA isolation, cDNA synthesis, and qRT-PCR

To conduct tissue-specific expression analysis, *S. alterniflora* seedlings were grown in an artificial chamber with a temperature of 24/22°C (day/night) and a photoperiod of 16 hours/8 hours (day/night). During the flowering stage, roots, stems, leaves, and inflorescences were collected for analysis. Immediately after sampling, the samples were rapidly frozen in liquid nitrogen and stored at -80°C for RNA extraction.

Total RNA was extracted from plants using the RNAsimple Total RNA Kit (Tiangen, Beijing) following the manufacturer’s instructions. The synthesis of the first-strand cDNA was performed using the FastKing RT Kit (Tiangen, Beijing), and the experimental steps from the kit’s manual were followed. qRT-PCR was conducted using the SYBR Green Pro Taq HS Kit (Tiangen, Beijing), following the manufacturer’s protocol. Data was normalized by the expression of *GAPDH* in *S. alterniflora* and *AtEF1α4* in *Arabidopsis.* Each group of experiments was repeated in three biological replicates, and the relative expression levels were determined using the 2^–ΔΔCt^ analysis method (Livak and Schmittgen, 2001). DNA extraction was carried out using the CTAB method. The primer sequences used in this study are listed in **Supplementary Table 1**.

### 2.5 *Arabidopsis* transformation and phenotypic analysis

The *SaTTG1* CDS was efficiently amplified by RT-PCR and cloned into the pCAMBIA-1300 vector. Transgenic *Arabidopsis* Columbia-0 lines were obtained using the floral dip method (Clough and Bent, 1998). Positive lines were screened on 1/2 MS solid medium with 40mg/L Hygromycin B and genotype verified by PCR. Mature seeds from wild-type and transgenic lines were imaged using an SMZ25 microscope (Nikon).

### 2.6 Subcellular localization and transcriptional activity assays

To ascertain the subcellular localization of SaTTG1, the CDS of *SaTTG1* was cloned into *PAN580*-GFP and introduced into rice (*Oryza sativa*) protoplasts. Subsequently, subcellular localization was observed using a confocal laser scanning microscope (Zeiss LSM 980 with Airysca, Germany). To assess transcriptional activity, the *SaTTG1* CDS was ligated into the *pGBKT7* vector for fusion expression. The resulting *pGBKT7*-*SaTTG1* was introduced into Y2H, which were then serially diluted 10-fold and spotted on SD/-Trp plates. Transcriptional activity was evaluated by monitoring yeast growth on SD/-Trp-Ade-His plates. The *pGBKT7* vector served as a negative control, with *pGBKT7*-*P53* as a positive control.

### 2.7 Statistical analysis

Root, seed length, and width of *Arabidopsis* were quantified with ImageJ (Rueden et al., 2017) and analyzed in Microsoft Excel 2013. Error bars represent standard deviation, and significant differences are marked with numbers or letters.

## 3 Results

### 3.1 The *S. alterniflora WD40* gene family

**Figure 1:**
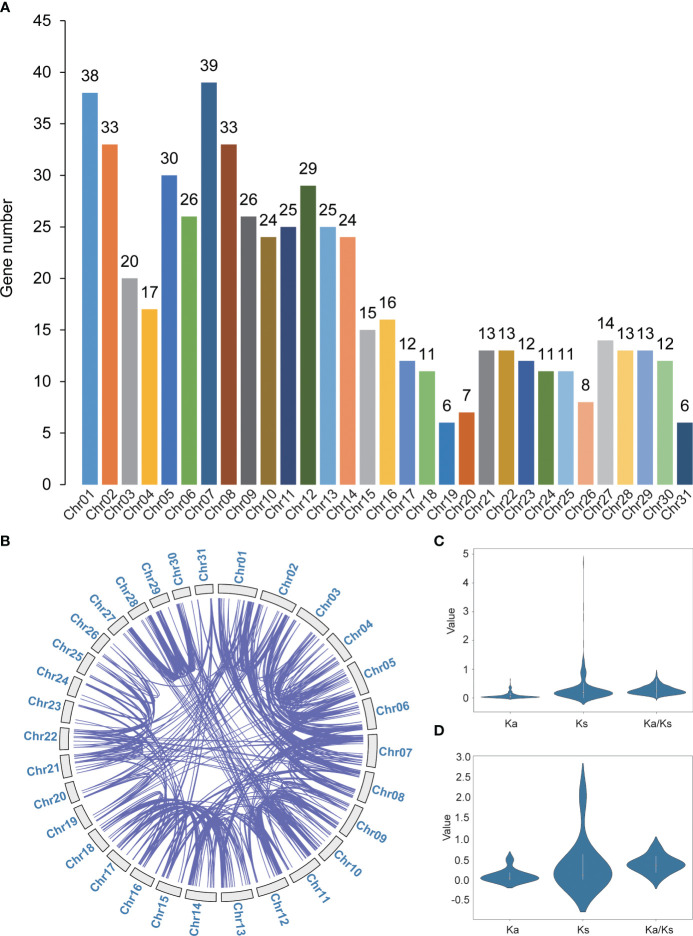
Identification and duplication analysis of the *S. alterniflora WD40* gene family. **(A)** Chromosomal distribution and **(B)** segmental duplication events of *SaWD40* genes. Connecting lines depict duplicated genes that together form pairs of duplicated genes. **(C)** The distribution values of ka, Ks, and Ka/Ks for segmental duplicate gene pairs. **(D)** The distribution values of ka, Ks, and Ka/Ks for tandem duplicate gene pairs. These values offer crucial insights into the selection pressures and evolutionary trends associated with these gene duplicates.

To unravel the functional aspects of the *WD40* gene family in *S. alterniflora*, we employed the conserved domain of WD40 proteins (PF00400) as a query in the Pfam database, conducting a comprehensive search against the *S. alterniflora* genome protein sequences. This exploration led to the identification of a total of 582 SaWD40 members distributed across 31 chromosomes. Chromosome 7 exhibited the highest density of *SaWD40* genes, while chromosomes 19 and 31 displayed the lowest density (**Figure 1A**). The nomenclature of *SaWD40* genes was assigned as *SaWD40–1* to *SaWD40–582* based on their respective chromosome locations (**Supplementary Table 2**). The deduced *SaWD40* DNA lengths ranged from 456 (*SaWD40–276*) to 52,866 bp (*SaWD40–311*), and protein lengths spanned from 70 (SaWD40–276) to 3,581 (SaWD40–30) amino acids (aa). The molecular weight varied between 7.44 (SaWD40–276) and 398.31 (SaWD40–30) kDa. Detailed physicochemical properties of *S. alterniflora* are comprehensively listed in **Supplementary Table 2**.

### 3.2 Gene duplication and phylogenetic analyses

Within the cohort of 582 *SaWD40* genes, 490 genes participated in the formation of 605 segmentally duplicated gene pairs, while 20 genes contributed to 10 tandemly duplicated gene pairs (**Figure 1B**; **Supplementary Table 3**). This implies a significant role of segmental duplication in expanding the *SaWD40* gene family. Further scrutiny of the synonymous (*Ks*) and non-synonymous (*Ka*) mutations in these gene pairs (**Figures 1C, D**; **Supplementary Table 3**) provided insights into the evolutionary dynamics. Moreover, the substitution rate (Ka/Ks) effectively determines positive selection pressure post-duplication, revealing evolutionary direction and selective strength in coding sequences. A Ka/Ks ratio of 1 indicates neutral selection, <1 purifying selection, and >1 positive selection. We calculated Ka, Ks, and Ka/Ks for each gene pair. The average Ka/Ks for tandem duplication *SaWD40* genes (0.3875) was significantly higher than for segmental duplication genes (0.2735). Additionally, tandem and segmental duplication events were estimated to have occurred ~256.85–357.58 and ~205.48–286.06 Mya, respectively. This observation suggests the prevalence of purifying selection, indicating that these genes have encountered functional constraints and selective pressure in maintaining their essential functions.

**Figure 2:**
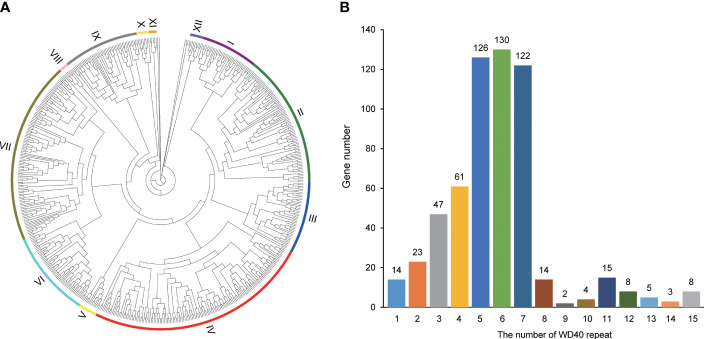
Phylogenetic and WD40 repeat analyses in *S. alterniflora.* **(A)** Phylogenetic analysis of WD40 proteins in *S. alterniflora*, constructed using the neighbor-joining method in MEGA 7. The tree exhibits 12 distinct phylogenetic clades with robust bootstrap support. **(B)** Summary of the number of WD40 repeats in *S. alterniflora*.

To depict the evolutionary relationships among these 582 SaWD40 proteins, we constructed an unrooted phylogenetic tree utilizing the NJ method (**Figure 2A**; **Supplementary Figure 1**). The bootstrap values within the tree facilitated the division of these WD40 proteins into 12 clades. Clade IV boasted the largest contingent of WD40 members, encompassing 145 members, followed by 118 in Clade VII and 87 in Clade II, whereas Clade XI exhibited a more modest presence with only five members.

### 3.3 Gene structure and protein composition analyses

The *SaWD40* genes exhibit a wide spectrum of WD40 repeats, ranging from 1 to 15 WD40 repeats (**Figure 2B**). Previous studies have indicated that proteins containing WD repeats typically possess 4–10 WD repeats (Van Nocker and Ludwig, 2003). In our SaWD40 protein repertoire, 459 members were identified to contain 4–10 WD repeats, while 39 members displayed more than 10 WD repeats.

We delved into the exon/intron structures of the *SaWD40* genes. The analysis unveiled a variation in the number of exons within the *SaWD40* gene structure, spanning from 1 to 41 (**Supplementary Table 2**), with an average of 10.83 exons. Remarkably, *SaWD40–311* boasted the largest number of exons (41), followed by *SaWD40–340* with 39 exons. In contrast, 21 *SaWD40* (3.6%) genes were observed to possess only one exon. The prevalence of genes with seven exons stood out, constituting the highest percentage at 8.8% (51/582) among the total gene count.

### 3.4 Expression patterns of *WD40* in different tissues

**Figure 3:**
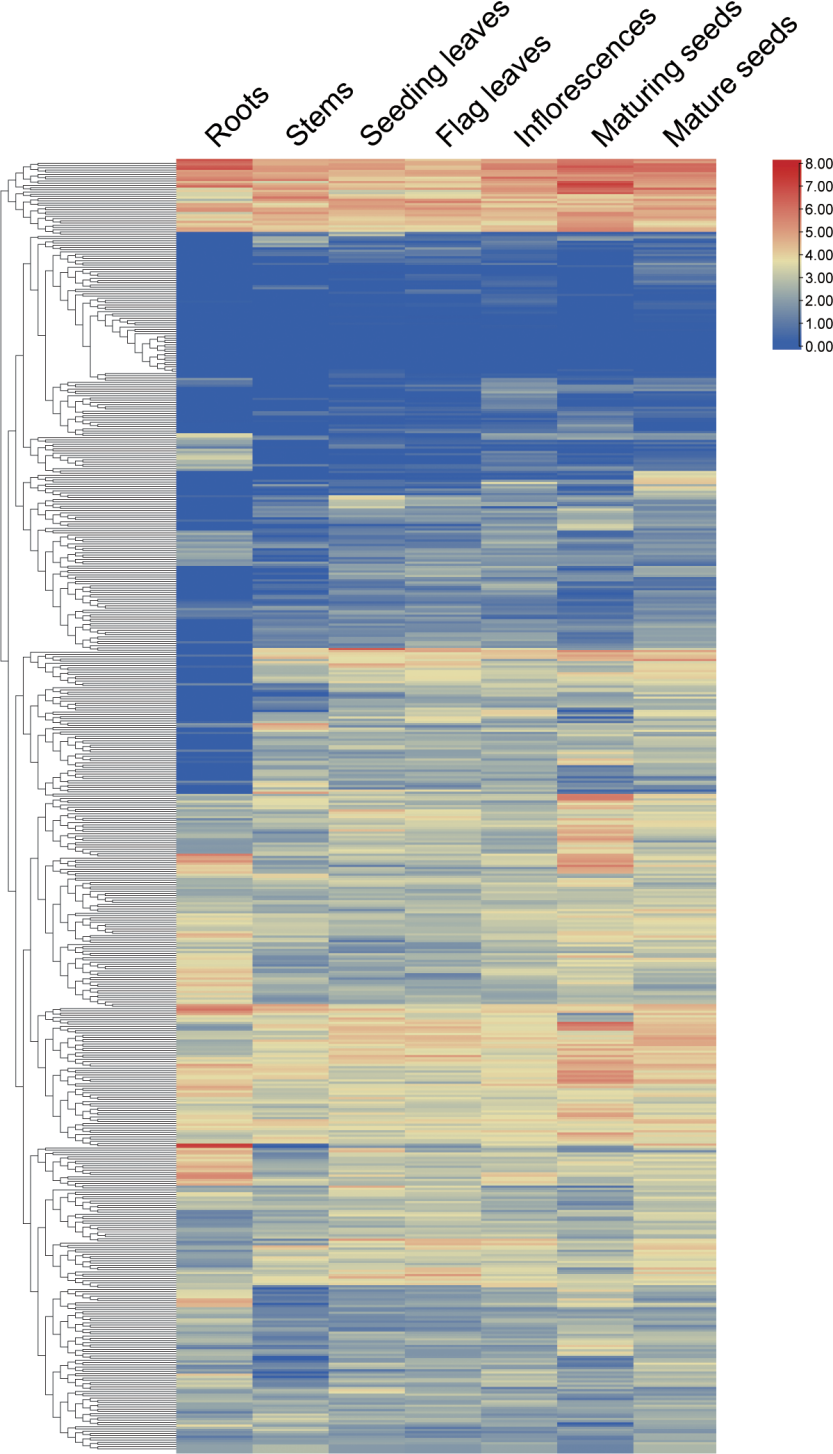
Expression patterns of *SaWD40* genes in various tissues using RNA-seq data. The color intensity reflects expression levels, with red indicating higher expression and blue indicating lower expression.

To elucidate the functional roles of *WD40* genes, we conducted an analysis of their expression patterns across various tissues in the *S. alterniflora* transcriptome. As depicted in **Figure 3** and detailed in **Supplementary Table 4**, with the exception of 11 genes (FPKM < 0.1) that exhibited undetectable expression levels across different tissues – namely *SaWD40–29*, *-74*, -*103*, -*152*, -*194*, -*220*, -*271*, -*345*, -*374*, -*392*, -*580* – the remaining 565 genes can be detected exhibiting expression (FPKM ≥ 0.1) in certain tissues. Three hundred sixty genes displayed ubiquitous expression across all tissues. There were nine genes that exhibit distinct tissue-specific expression patterns. For instance, *SaWD40–194* exhibited exclusive expression in roots, while *SaWD40–346, SaWD40–379*, *SaWD40–391*, and *SaWD40–502* were specifically expressed in developing inflorescences; *SaWD40–85*, *SaWD40–193*, *SaWD40–376*, and *SaWD40–500* displayed specific expression in mature seeds. Furthermore, we observed that certain genes are expressed at higher levels in specific tissues compared to other tissues. For instance, eight genes (*SaWD40–73*, *SaWD40–252*, *SaWD40–256*, *SaWD40–293*, *SaWD40–258*, *SaWD40–283*, *SaWD40–444*, *SaWD40–477*) exhibited higher expression levels in maturing seeds, while eight genes (*SaWD40–19*, *SaWD40–102*, *SaWD40–107*, *SaWD40–125*, *SaWD40–219*, *SaWD40–272*, *SaWD40–488*, *SaWD40–514*) demonstrated higher expression in developing inflorescences. These findings underscore the relatively widespread expression of *WD40* genes across diverse tissues.

### 3.5 Protein structure and expression patterns of *SaTTG1*

Analysis of expression patterns revealed that *WD40* genes can be detected in various tissues of *S. alterniflora*, indicating their involvement in diverse growth and development stages of the plant. To validate the role of *WD40* genes in plant growth and development, we aimed to functionally characterize them through heterologous expression in *A. thaliana*. *TTG1*, a gene encoding a WD40 protein, has been shown to play a pivotal role in multiple aspects of plant growth and development. Its involvement spans processes such as the accumulation of seed storage reserves (Chen et al., 2015), biosynthesis of anthocyanin and proanthocyanidin (Shan et al., 2019), regulation of circadian activity, epidermal cell fate, and pigmentation (Airoldi et al., 2019). However, to date, there have been no reports on the functional roles of *WD40* genes in *S. alterniflora*. Expression pattern analysis revealed that *SaWD40–256*, a homolog of *A. thaliana*’s *TTG1* gene, is expressed at its highest level in maturing seeds. Therefore, we aimed to functionally characterize the *S. alterniflora TTG1* gene through heterologous expression in *A. thaliana*, with the goal of gaining deeper insights into the conservation of its functional properties.

**Figure 4:**
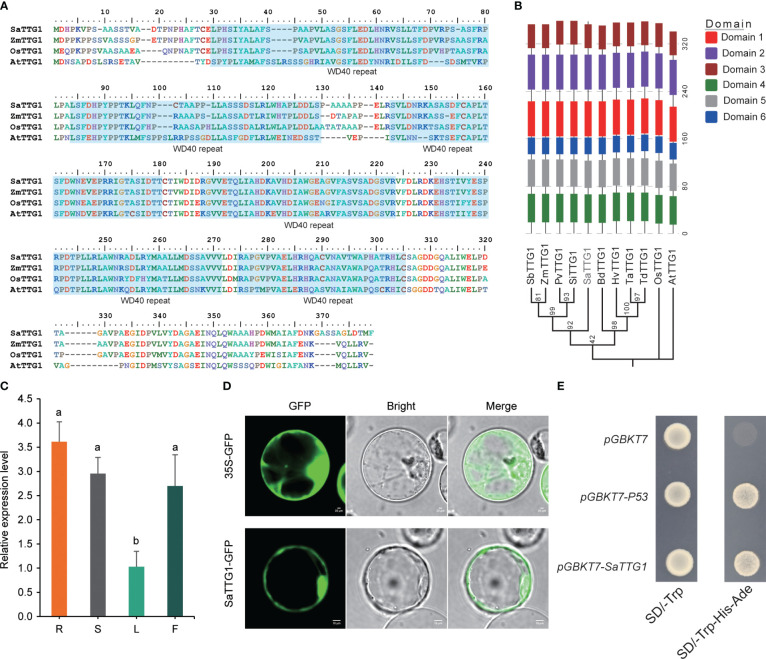
Protein structure and expression patterns of *SaTTG1*. **(A)** Sequence alignment of TTG1 proteins from *S. alterniflora*, *Arabidopsis*, rice, and maize. The blue shaded area indicates WD40 repeats. **(B)** Phylogenetic and conserved domain analyses of TTG1 proteins from monocot and dicot plants. *Sorghum bicolor* SbTTG1, AFN17366.1; *Zea mays* ZmTTG1, NP_001310302.1; *Panicum virgatum* PvTTG1, XP_039854481.1; *Setaria italica* SiTTG1, XP_004953461.1; *Brachypodium distachyon* BdTTG1, XP_003570109.1; *Hordeum vulgare* HvTTG1, XP_044952062.1; *Triticum aestivum* TaTTG1, XP_044403847.1; *Triticum dicoccoides* TdTTG1, XP_037447801.1; *Oryza sativa* OsTTG1, NP_001403759.1; *Arabidopsis thaliana* AtTTG1, CAC10524.1. **(C)** qRT-PCR analysis of *SaTTG1* in various tissues. R, S, L, and F represent the root, stem, leaf, and inflorescence tissues of *S. alterniflora*, respectively. The values presented are expressed as means ± SD, with three biological replicates (n=3). Significant differences at *P* < 0.05 were determined using Duncan’s multiple range test, and are indicated by different letters. **(D)** Subcellular localization of the SaTTG1 protein fused with eGFP in rice protoplasts. Scale bar = 20 μm. **(E)** Transcriptional activity of SaTTG1 in yeast cells. Yeast cells containing *pGBKT7*-*P53* were utilized as a positive control, while yeast cells containing *pGBKT7* empty vector served as a negative control.

Seeking a comprehensive understanding of the function of *SaTTG1* (*SaWD40–256*) in *S. alterniflora*, we isolated the gene from young seedlings of this species. As depicted in **Figure 4A**, SaTTG1 exhibits a high sequence similarity with TTG1 proteins from *Arabidopsis*, *Zea mays*, and *Oryza sativa*. These proteins share a highly conserved WD40 repeat regulatory domain. To unravel the potential functions of the SaTTG1 protein, a phylogenetic analysis was conducted using SaTTG1 and TTG1 proteins from other plants. The results suggest that the functions of SaTTG1 more closely resemble those of monocot proteins than dicot proteins (**Figure 4B**; **Supplementary Figure 2**).

**Figure 4C** illustrates the presence of *SaTTG1* transcript in various tissues, with the lowest expression level detected in leaves. This indicates that the gene plays a widespread role in the growth and developmental processes of plants. Subcellular localization studies revealed that *SaTTG1*, when transiently expressed as 2×35S::*SaTTG1*-eGFP in rice protoplasts, exhibited a fluorescent signal exclusively in the nucleus and plasma membrane (**Figure 4D**). Furthermore, yeast cells transformed with the pGBDT7-*SaTTG1* fusion construct demonstrated activation of reporter genes and survival in the selective medium SD/-Trp-Ade-His (**Figure 4E**), affirming the transcriptional activation activity of SaTTG1 in yeast.

### 3.6 Overexpression of *SaTTG1* in *Arabidopsis* modulates plant development

**Figure 5:**
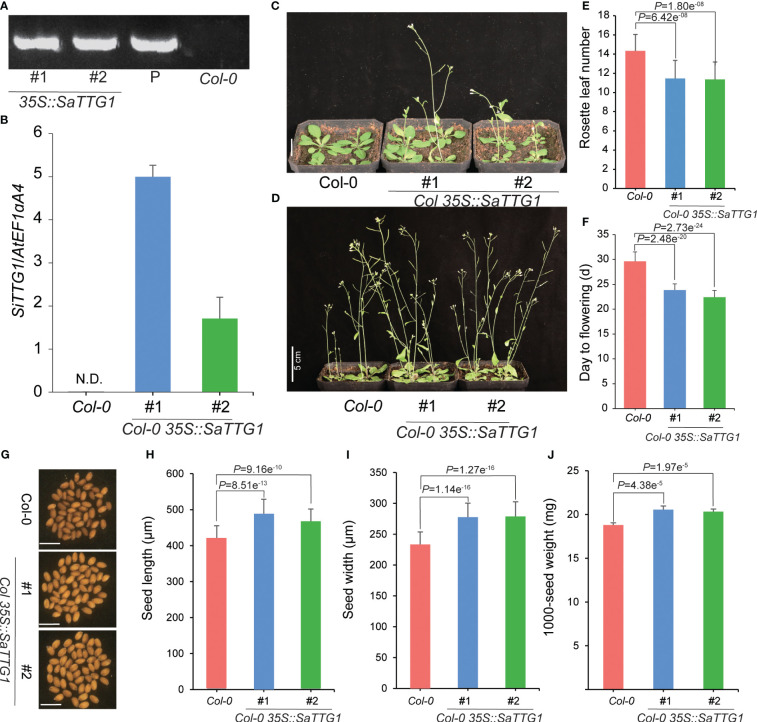
Overexpression of *SaTTG1* in *Arabidopsis* regulates plant development. **(A)** PCR-based DNA genotyping of the transgenic lines using specific primers. **(B)** Relative expression levels of the transgenic lines assessed by qRT-PCR, normalized against the expression of the internal control *AtEF1α;A4*. **(C, D)** Phenotypes of Col-0 and transgenic lines at 21 **(C)**, Scale bar = 1 cm) and 28 days **(D)**, Scale bar = 5 cm), respectively. **(E)** Statistical analysis of the number of rosette leaves on the 28th day. **(F)** Flowering time statistics. **(G–J)** Seed phenotypes of Col-0 and transgenic lines, including **(G)** seed phenotype photo (Scale bar = 1 mm), **(H)** Seed length statistics (n = 30), **(I)** Seed width statistics (n = 30), and **(J)** 1000-seed weight statistics (n = 6). Error bars represent the standard deviation, and significance tests were conducted using Student’s *t*-tests.

To examine the function of *SaTTG1*, we introduced the overexpression construct of 35S::*SaTTG1* into the *Arabidopsis* wild-type Col-0. Through hygromycin selection, a total of 20 independent T1 transgenic plants were acquired. Subsequently, two independent T3 homozygous Col-0 *35S*::*SaTTG1* transgenic lines (#1 and #2) were selected and validated by PCR (**Figure 5A**). qRT-PCR analysis corroborated the substantial expression of *SaTTG1* in the respective transgenic lines, with no detection in the wild-type Col-0 (**Figure 5B**). These results demonstrate that the overexpression of *SaTTG1* induces early flowering in *Arabidopsis*, and the number of rosette leaves in overexpressing plants is reduced (**Figures 5C–F**). The seed size of the transgenic lines surpassed that of Col-0, displaying augmented seed length and width (**Figures 5G–I**). Correspondingly, the 1000-seed weight of the transgenic lines exhibited a notable increase by 9.29% and 8.10% compared to Col-0 (**Figure 5J**). These findings underscore the significant role of *SaTTG1* in the regulation of plant development.

## 4 Discussion

*WD40* constitutes a substantial gene family identified in various plant species, encompassing *Arabidopsis*, wheat, *Flammulina velutipes*, *Cucurbita maxima*, and others (Van Nocker and Ludwig, 2003; Hu et al., 2018; Chen et al., 2023; Ji et al., 2023). Our investigation discerned a higher count of *WD40* genes in *S. alterniflora* compared to other species, implying potential gene duplication events during evolution. In *S. alterniflora*, 490 *SaWD40* genes formed 605 segmentally duplicated gene pairs, and 20 *SaWD40* genes arranged into 10 tandemly duplicated gene pairs, reinforcing the notion that gene duplication has substantially contributed to the proliferation of *WD40* genes. A meticulous analysis of *Ka*/*Ks* values for all duplicated gene pairs disclosed that the *Ka*/*Ks* ratio predominantly remained at less than 1, indicating of the prevailing influence of purifying selection on these genes.

Diversity in gene structure, denoting variations in gene sequences within a species, resulting in genetic polymorphism, was evident in *SaWD40*. The gene structure composition displayed considerable diversity, with the number of exons ranging from 1 to 41. Comparable diversity is witnessed in the gene structures of other plants. For instance, wheat *WD40* genes exhibit a range of 1 to 39 exons (Hu et al., 2018), *Cerasus humilis* genes vary from 1 to 51 (Ji et al., 2023), and *Cucurbita maxima* genes showcase between 1 and 30 exons (Chen et al., 2023). This observation underscores the widespread distribution of *WD40* across different species, indicative of the functional diversification of *WD40* genes. Moreover, the uneven distribution of WD40 domains in the SaWD40 protein contributes to further nuances in the functional diversification of *SaWD40* genes.

The *WD40* gene stands as a pivotal player in plant growth and development, with *TTG1* being a focal point in numerous studies, highlighting its predominant expression in tissues with anthocyanin accumulation. In this study, we observed the presence of *SaTTG1* across different tissues, with the lowest expression level detected in leaves. The relatively low expression level of *SaTTG1* in leaves may suggest that its function in this tissue is not dominant or may differ from its roles in other tissues. Diverse plant species exhibit tissue-specific expression patterns of *TTG1*, such as apple *TTG1* primarily expressed in the peel (An et al., 2012), Chinese bayberry *TTG1* gene highly expressed in the fruit (Liu et al., 2013), and *Freesia*×*hybrida TTG1* mainly expressed in the petals (Shan et al., 2019). In *Arabidopsis*, *TTG1* plays a crucial role in flowering time regulation (Paffendorf et al., 2020). Our observations align with these findings, demonstrating that overexpression of the *SaTTG1* gene in *Arabidopsis* induces early flowering. The tissue-specific expression patterns observed across different plant species also highlight the specificity of *TTG1*’s functions in plant development. Overall, our results contribute to the understanding of the diverse roles of *TTG1* in plant growth and development, particularly in relation to flowering time regulation.

Moreover, *TTG1* emerges as a critical player in seed development, occupying a strategic position in the regulatory hierarchy governing seed filling. Directly targeted by FUS3, *TTG1* regulates the accumulation of seed storage proteins and fatty acids during seed maturation (Chen et al., 2015). Overexpression of *Setaria italica SiTTG1* has been shown to rectify reduced expression of mucilage biosynthetic genes, including genes involved in seed fatty acid and storage protein accumulation in *ttg1–13* plants (Liu et al., 2017). Our research substantiates these roles, indicating that overexpression of *SaTTG1* in *Arabidopsis* significantly influences seed size, underscoring *TTG1* as a critical determinant in plant seed development.

Genes from industrial crops, such as switchgrass (*Panicum virgatum*), sugarcane (*Saccharum officinarum*), and cassava (*Manihot esculenta*), possess the potential to enhance the energy yield of plants through modification of growth and development processes. Overexpression of specific genes, such as switchgrass *PvBiP2* and *PvWOX3a* in switchgrass, can increase biomass yield and enhance stem development, respectively (Yang et al., 2021; Song et al., 2023). In sugarcane, *SNF4* and its sorghum ortholog *SNF4* impact biomass and sugar yield (Upadhyaya et al., 2022), while overexpression of the *SoSPS1* gene enhances sucrose content (Anur et al., 2020). Similarly, in cassava, overexpression of *MeSLAH4* enhances nitrogen assimilation, growth, and yield (Song et al., 2022). These findings highlight the potential of industrial crops for enhancing energy production through genetic manipulation. As a halophyte, *S. alterniflora* has the characteristics of industrial and energy crops (Buhle et al., 2012). In this study, analysis of gene expression patterns revealed that a total of 360 *WD40* genes are expressed in various tissues, with many genes exhibiting tissue-specific expression patterns. Overexpression of the *SaTTG1* gene can alter seed size and weight, indicating its potential for improving seed yield. This finding suggests that the gene can also be used for genetic improvement in industrial crops.

## 5 Conclusion

In this investigation, we systematically identified 582 *WD40* genes within the genome of *S. alterniflora*, showcasing an uneven distribution across the species’ 31 chromosomes. The expansion of *SaWD40* genes is attributed to gene duplication events, introducing diversity in the composition of gene structures. SaTTG1, a member of the WD40 family, is localized in both the nucleus and plasma membrane, demonstrating transcriptional activation activity. When introduced into *Arabidopsis*, *SaTTG1* significantly modifies the flowering time and seed size of the transgenic plants. Our study sheds light on the multifaceted functions of *WD40* genes, laying the groundwork for further exploration of their intricate roles in the realm of plant biology.

## Data availability statement

The datasets presented in this study can be found in online repositories. The names of the repository/repositories and accession number(s) can be found in the article/**Supplementary Material**.

## Author contributions

MY: Formal analysis, Investigation, Visualization, Writing – original draft. SKC: Formal analysis, Investigation, Visualization, Writing – original draft. JG: Data curation, Investigation, Validation, Writing – review & editing. SG: Data curation, Investigation, Validation, Writing – review & editing. SHC: Funding acquisition, Supervision, Validation, Writing – review & editing. HL: Conceptualization, Funding acquisition, Resources, Supervision, Writing – review & editing.
