# Supplementary material for: Comprehensive analysis of the Spartina alterniflora WD40 gene family reveals the regulatory role of SaTTG1 in plant development
Source: Front Plant Sci. 2024 May 28;15:1390461. doi: 10.3389/fpls.2024.1390461 (PMC11165199; doi:10.3389/fpls.2024.1390461)
Supplement: Supplementary file 1 [file DataSheet_1.pdf]

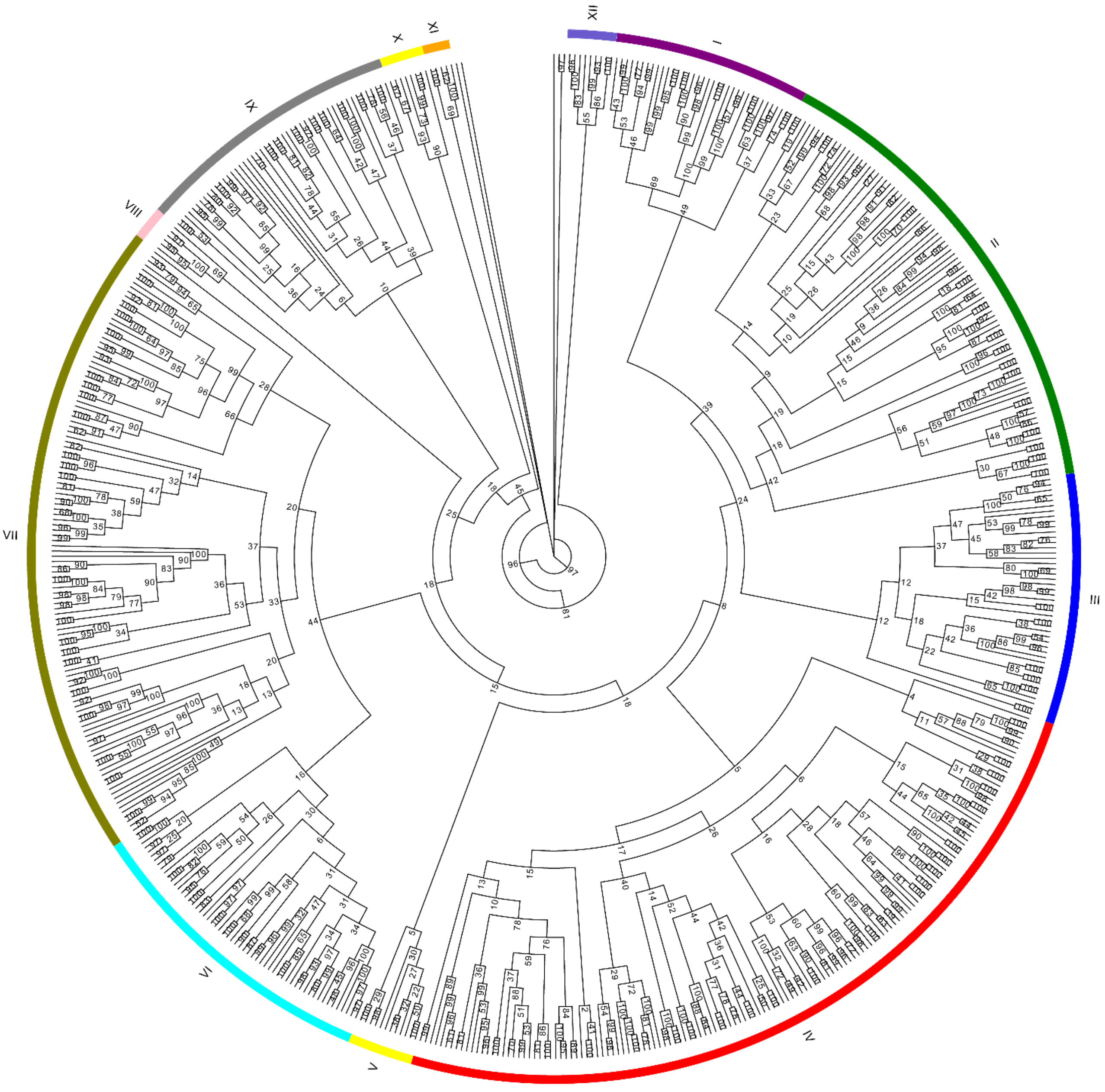

Figure S1 Phylogenetic and WD40 repeat analyses in *S. alterniflora*.

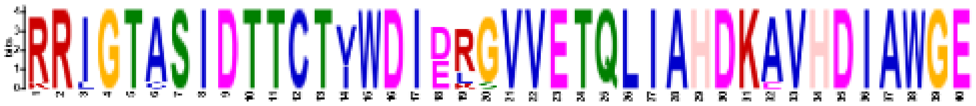

Domain 1 RRIGTASIDTTCT[VI]WDI[DE]RGVVETQLIAHDKAVHDIAWGE

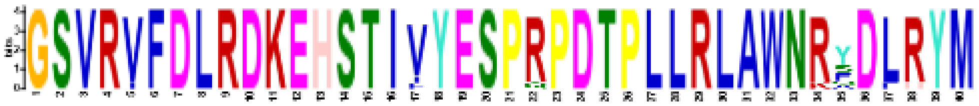

Domain 2 GSVRVFDLRDKEHSTIVYESPRPDTPLLRLAWNRYDLRYM

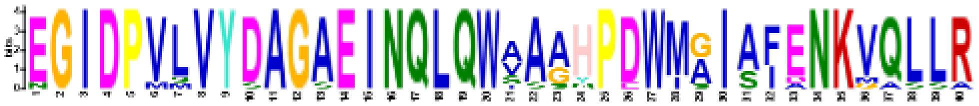

Domain 3 EGIDPVLVYDAGAEINQLQW[AV]A[AG]HPDWM[AG]I[AS][FI]ENKVQLLR

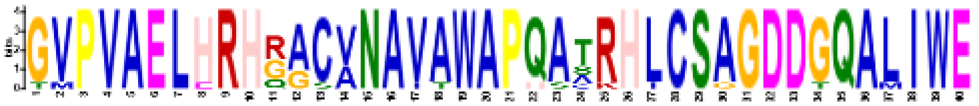

Domain 4 GVPVAELHRH[RG][AG]C[VA]NAVAWAPQATRHLCSAGDDGQALIWE

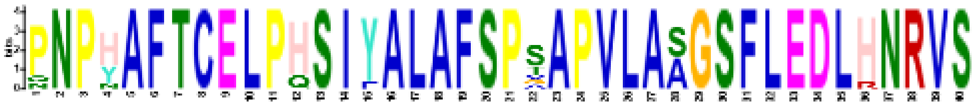

Domain 5 PNP[HY]AFTCELP[HQ]SIYALAFSP[ST]APVLA[AS]GSFLEDLHNRVS

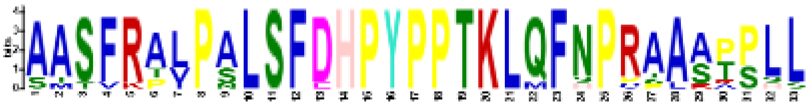

Domain 6 AASFRA[LV]P[AS]LSFDHPYPPTKLQFNPRAA[AS][PT][PS]LL

Figure S2 Regulator expression of six conserved domains
